# Supplementary material for: A trans-synaptic IgLON adhesion molecular complex directly contacts and clusters a nicotinic receptor
Source: Nat Commun. 2026 Jan 22;17:1404. doi: 10.1038/s41467-025-68141-1 (PMC12881522; doi:10.1038/s41467-025-68141-1)
Supplement: Supplementary file 1 — Supplementary Information [file 41467_2025_68141_MOESM1_ESM.pdf]

Supplementary information for:

A trans-synaptic IgLON adhesion molecular complex directly contacts and clusters a nicotinic receptor

a

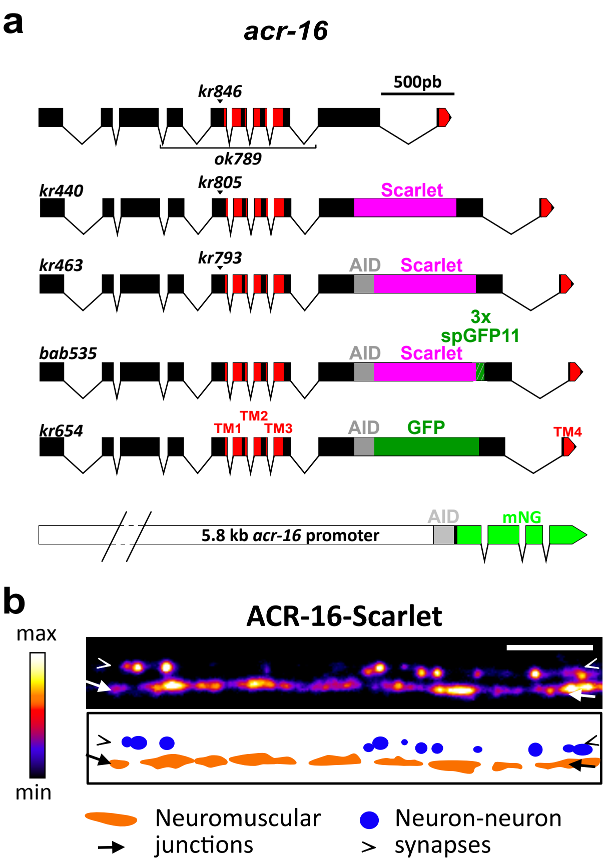

c

Neurons expressing ACR-16	Expression of the *acr-16::aid::mNG* transcriptional reporter	Neurite in VNC	Synapses in VNC	Expression of *rig-5*	Expression of *zig-8*
AVA	yes	yes	yes	yes	yes
DB	yes	yes	yes	no	yes
AVB	no	yes	yes	no	no
DA	no	yes	yes	no	yes
PVR	yes	yes	no	no	no
RIB	yes	no		yes	no
SMD	yes	no		no	yes
M4	no	no		no	no
RIP	no	no		no	yes
SIA	no	no		no	no
M1	no	no		no	no
SIB	no	no		no	yes
RID	yes	no		no	no

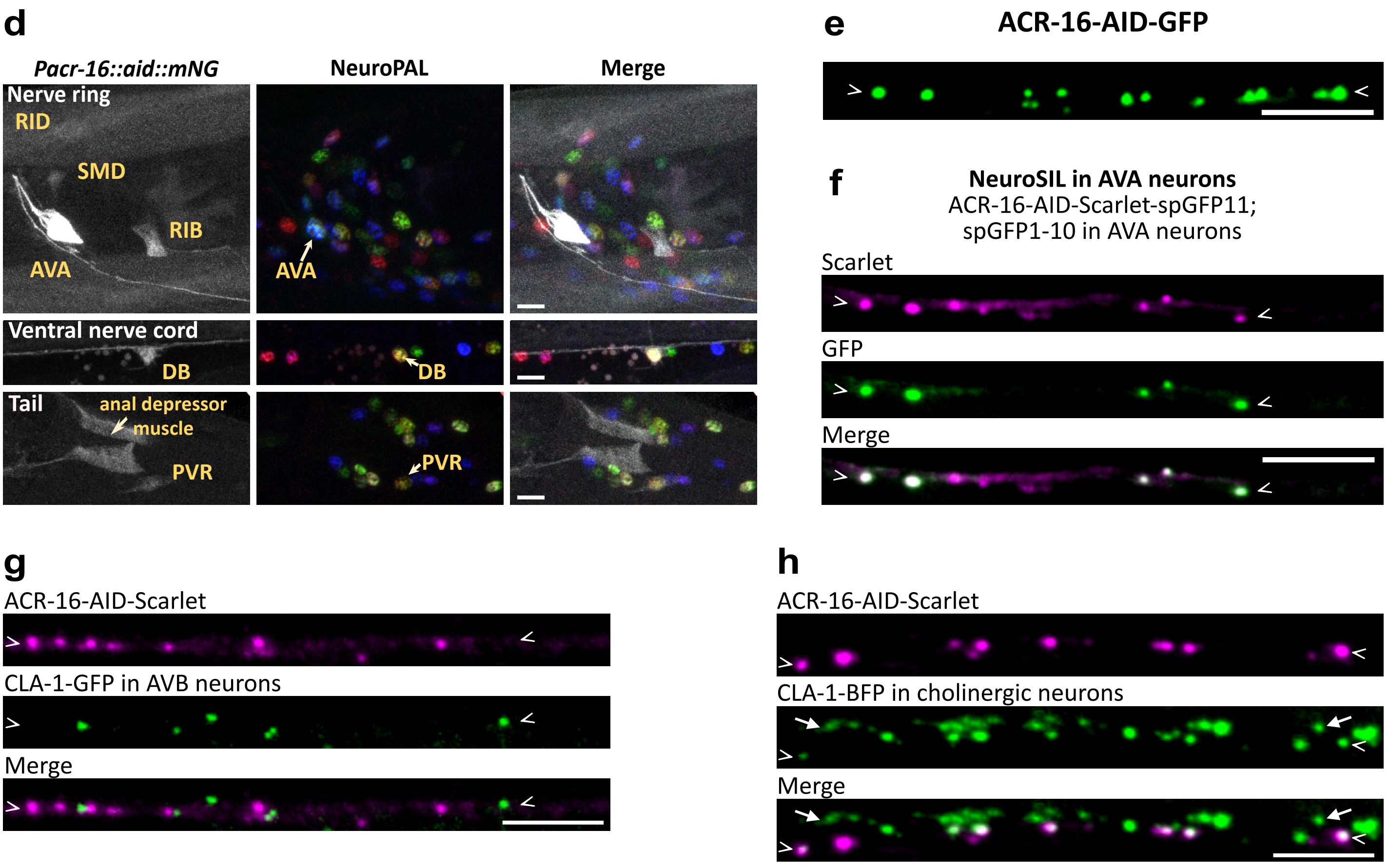

Supplementary Figure 1 (related to Figure 1). Analysis of ACR-16 expression and synaptic localization. (a) Genomic organization of the *acr-16* locus (boxes represent exons with sequences of the four transmembrane domains colored in red), and the positions of different tags and mutants used in this study (arrowheads represent point mutations; the bracket represents a deletion). Bottom: transcriptional reporter. Scale bar: 500 bp. **(b)** Spinning disk microscopy images and schematic of ACR-16-Scarlet fluorescence along the ventral cord at NMJs (orange dots, arrows) and neuron-neuron synapses (blue dots, arrowheads). **(c)** Table depicting neurons that display *acr-16* expression, whether they form neurites and synapses in the ventral nerve cord, and whether they express *rig-5* and *zig-8.* Expression data has been extracted from the CeNGEN atlas using a threshold of 2 (yes = detected with a threshold of 2, no = not detected). Connectivity data from White *et al.*, 1986. **(d)** The *acr-16* transcriptional reporter (*krIs83[Pacr-16::aid::mNG]*) was analyzed in the NeuroPAL background in the head, ventral nerve cord and tail, following auxin-induced degradation in muscle. **(e)** Punctate pattern of ACR-16-AID-GFP. **(f)** NeuroSIL images in AVA neurons showing Scarlet fluorescence and GFP reconstruction (ACR-16-AID-Scarlet-spGFP11, spGFP1-10 in AVAs). For (E-F), the puncta number was assessed in Figure 1C. **(g-h)** Endogenous ACR-16-AID-Scarlet, a CLA-1-GFP presynaptic reporter expressed in AVB neurons (g), and CLA-1-BFP in cholinergic neurons (h). Scale bars: 5 µm. Arrowheads and arrows delineate regions of the ventral nerve cord where neuron-neuron synapses and neuromuscular junctions, respectively, form.

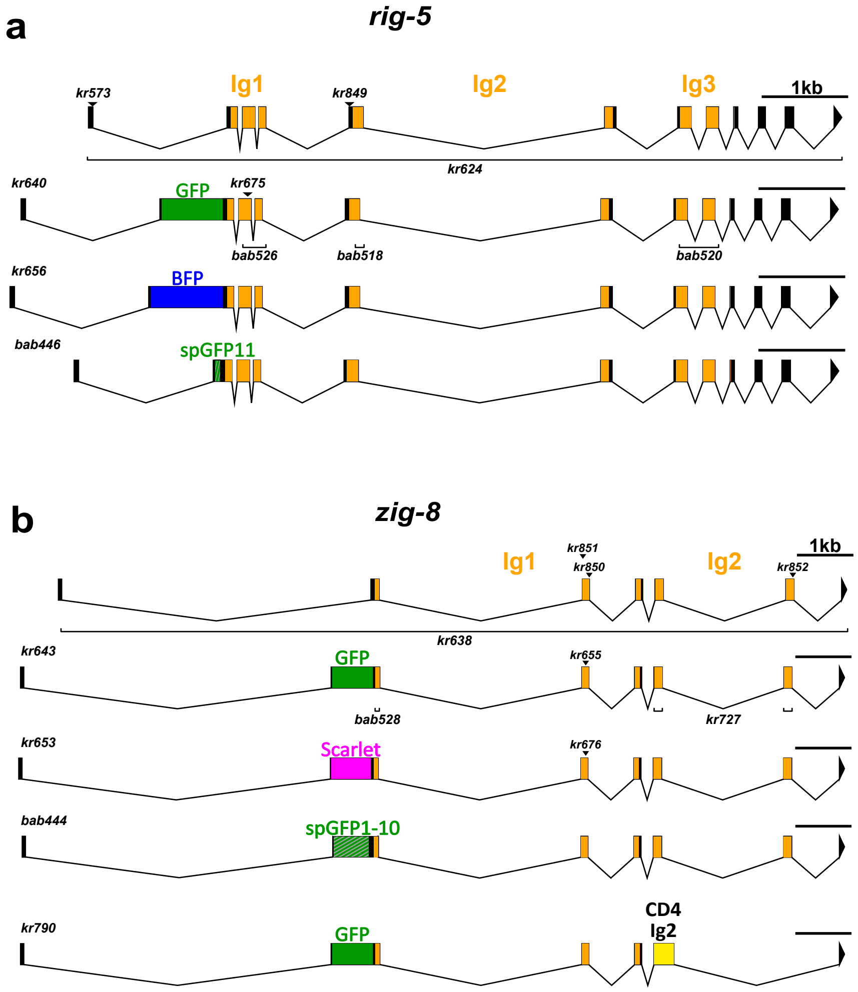
a
rig-5
Ig1
Ig2
Ig3
1kb
kr573
kr849
kr624
kr640
GFP
kr675
bab526
bab518
bab520
kr656
BFP
bab446
spGFP11
b
zig-8
Ig1
Ig2
1kb
kr851
kr850
kr852
kr638
kr643
GFP
kr655
bab528
kr727
kr653
Scarlet
kr676
bab444
spGFP1-10
kr790
GFP
CD4
Ig2

Supplementary Figure 2 (related to Figure 2). The *rig-5* and *zig-8* loci. (a) Genomic organization of *rig-5* and the positions of different tags and mutants used in this study. We used the longest isoform (C36F7.4e.1), considering the second methionine in the transcript as a translation start site, as it generates a strongly predicted signal peptide (see Supplementary Table S7 for sequence numbering and reference[1]). **(b)** *zig-8* locus, tags and mutants, including an insertion used in this study. Genome annotation of *C. elegans* reports a single transcript for *zig-8* (Y39E4B.8.1), which encodes a protein with UniProt accession number G5ED00 (Supplementary Table S7). Boxes represent exons with sequences of the Ig domains colored in orange. Ig domains were identified using the SMART web-based tool (Simple Modular Architecture Research Tool, http://SMART.embl-heidelberg.de). Complete coding sequence were deleted for the *rig-5* Ig1 and Ig3 domains (*rig-5(bab526)* and *rig-5(bab520)* alleles). In contrast, only partial deletions were created for the *rig-5* Ig2 and *zig-8* Ig1 domains due to the presence of large introns (*rig-5(bab518)* and *zig-8(bab528)* alleles). Additionally, a small portion of the coding sequence in the *zig-8* Ig2 domain was retained because of CRISPR design constraints (*zig-8(kr727)* allele). Arrowheads: point mutations; brackets: deletions. Supplementary Table S2 contains a brief description of *rig-5* and *zig-8* alleles, including deletion boundaries.

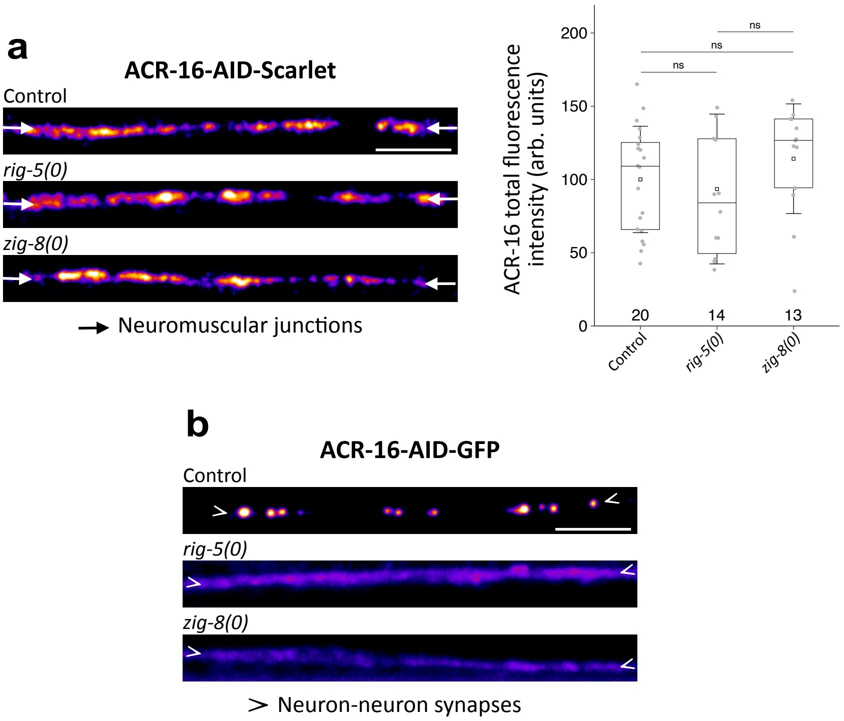
a
ACR-16-AID-Scarlet
Control
rig-5(0)
zig-8(0)
Neuromuscular junctions
ACR-16 total fluorescence intensity (arb. units)
200
150
100
50
0
ns
ns
ns
20
14
13
Control
rig-5(0)
zig-8(0)
b
ACR-16-AID-GFP
Control
rig-5(0)
zig-8(0)
Neuron-neuron synapses

Supplementary Figure 3 (related to Figure 3). *rig-5* and *zig-8* mutations prevent ACR-16 clustering at neuron-neuron synapses but not at neuromuscular junctions. (a) ACR-16-AID-Scarlet labelling at neuromuscular synapses of the dorsal nerve cord, with quantification of the total fluorescence intensity in a control strain and *rig-5(0)* and *zig-8(0)* mutants. **(b)** ACR-16-AID-GFP fluorescence shows clustering defects in neurons of the *rig-5(0)* and *zig-8(0)* mutants following auxin-mediated degradation in muscle cells. Data are presented as boxplots showing lower and upper quartiles (box), mean (square), median (center line) and standard deviation (whiskers); the number of worms is indicated for each condition; Kruskal-Wallis and Dunn's test (a); ns: non-significant. Scale bars: 5 µm. Arrowheads and arrows delineate regions of the ventral nerve cord where neuron-neuron synapses and neuromuscular junctions, respectively, may form.

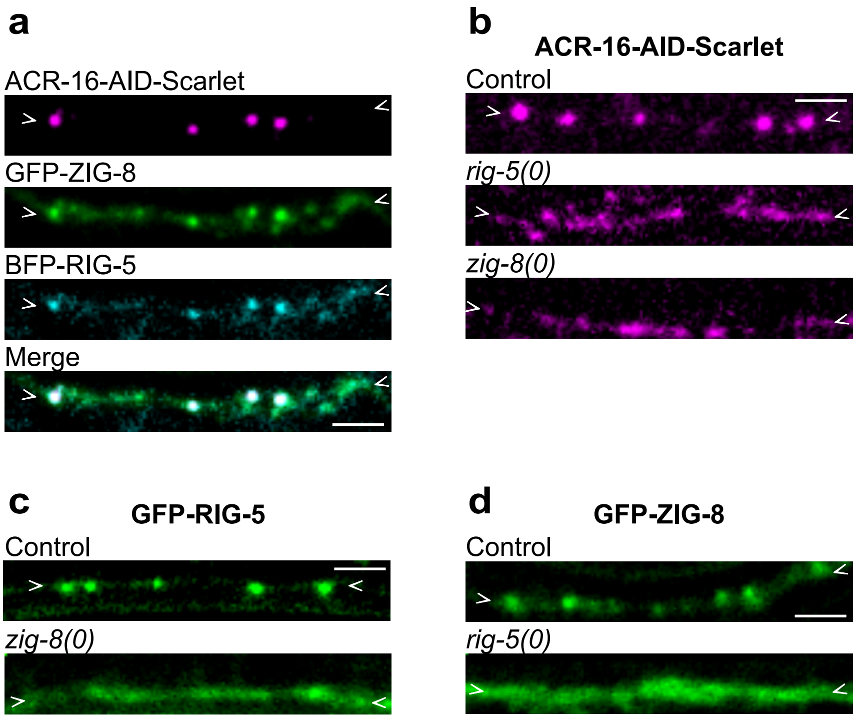

a
ACR-16-AID-Scarlet
GFP-ZIG-8
BFP-RIG-5
Merge
b
ACR-16-AID-Scarlet
Control
rig-5(0)
zig-8(0)
c
GFP-RIG-5
Control
zig-8(0)
d
GFP-ZIG-8
Control
rig-5(0)

Supplementary Figure 4 (related to Figure 3). RIG-5 and ZIG-8 control ACR-16 receptor clustering in L1 larvae. (a) Knock-in reporters of BFP-RIG-5 and GFP-ZIG-8 show strong colocalization with neuronal ACR-16-AID-Scarlet. **(b)** ACR-16-AID-Scarlet fluorescence shows clustering defects in neurons of the *rig-5(0)* and *zig-8(0)* mutants. **(c)** GFP-RIG-5 observed in a control strain and in the *zig-8(0)* mutant. **(d)** GFP-ZIG-8 observed in a control strain and in the *rig-5(0)* mutant. In this figure, all spinning disk microscopy images were taken in newly born L1 animals. For b, c and d, fluorescence intensities were enhanced in the *rig-5(0)* and *zig-8(0)* mutants. Scale bars: 2 µm. Arrowheads delineate neurites of the ventral nerve cord where synapses may form.

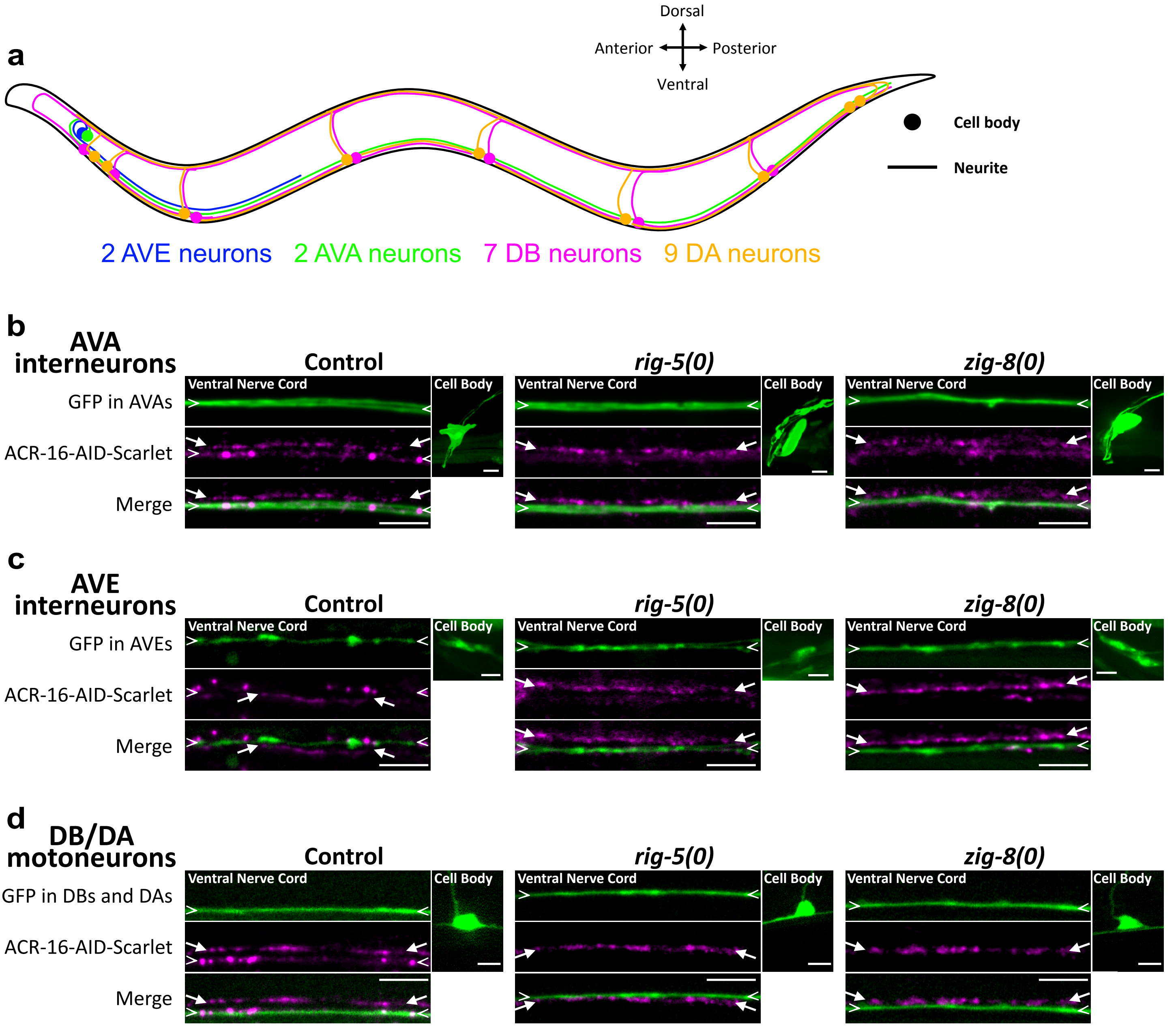

e

	AVA interneurons			AVE interneurons			DB/DA motoneurons		
Genotype	Number of worms	Number of cell bodies (2)	Guidance	Number of worms	Number of cell bodies (2)	Guidance	Number of worms	Number of cell bodies (16)	Guidance
Control	6	2	100%	17	2	100%	15	10-13	100%
rig-5(0)	6	2	100%	8	2	100%	11	10-13	100%
zig-8(0)	6	2	100%	17	2	100%	15	8-13	100%

Supplementary Figure 5 (related to Figure 3). ***rig-5*** **and** ***zig-8*** **mutations do not alter neuronal specification, migration or axon guidance of AVA, AVE and DB neurons. (a)** Schematic of cell bodies and neurites of AVE and AVA neurons, and DB- DA-types motoneurons (comprising the 7 DB-type and 9 DA-type neurons) in an adult *C. elegans*. **(b-d)** Assessment of the presence and position of cell bodies and neurites using cytoplasmic GFP specifically expressed in AVA (b), AVE (c) neurons and DB- DA-types motoneurons (d) in control backgrounds and in *rig-5(0)* and *zig-8(0)* mutants. Scale bars: 5 µm. **(e)** Summary table. Arrowheads and arrows delineate regions of the ventral nerve cord where neuron-neuron synapses and neuromuscular junctions, respectively, may form.

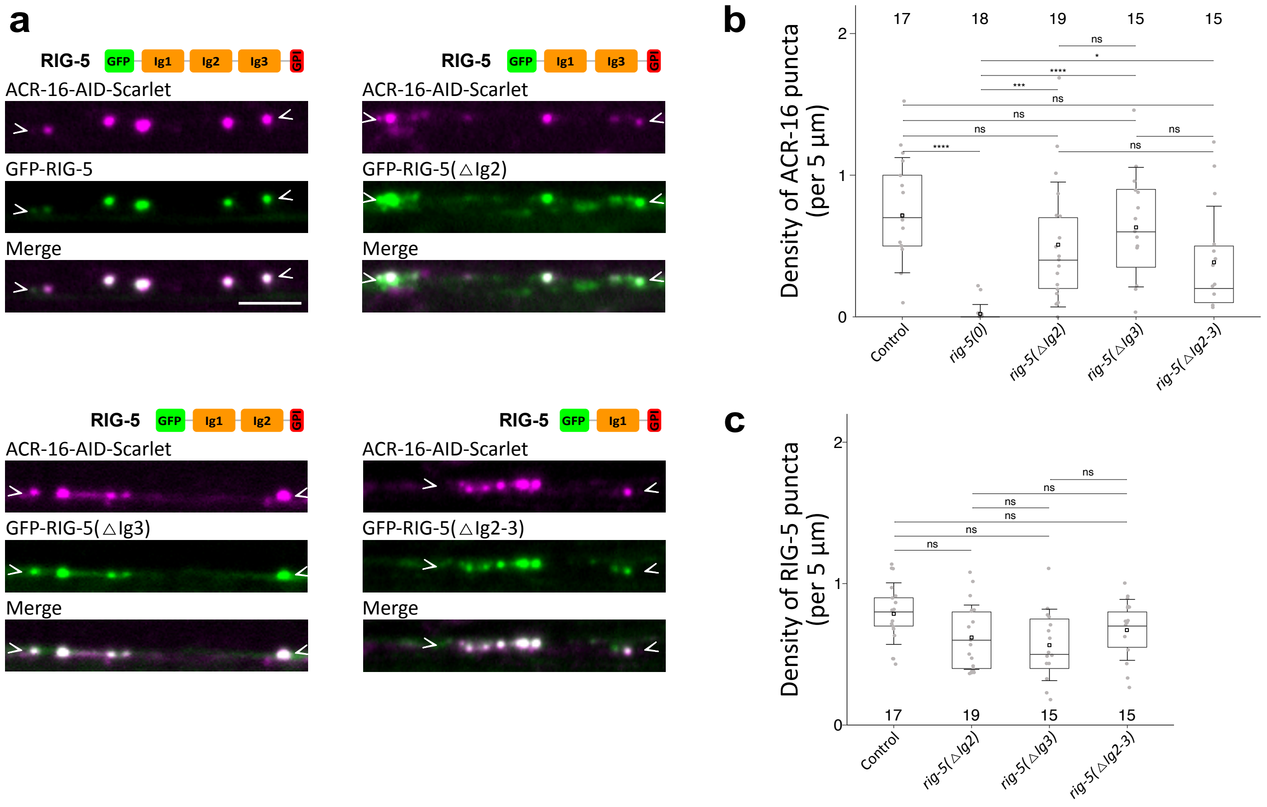
a
RIG-5
GFP
Ig1
Ig2
Ig3
GPI
ACR-16-AID-Scarlet
GFP-RIG-5
Merge
GFP-RIG-5(△Ig2)
GFP-RIG-5(△Ig3)
GFP-RIG-5(△Ig2-3)
b
Density of ACR-16 puncta (per 5 µm)
17
18
19
15
15
ns
*
****
***
****
Control
rig-5(0)
rig-5(△Ig2)
rig-5(△Ig3)
rig-5(△Ig2-3)
c
Density of RIG-5 puncta (per 5 µm)
17
19
15
15

Supplementary Figure 6 (related to Figure 5). RIG-5 Ig2 and Ig3 domains are dispensable for ACR-16 receptor clustering. (a) ACR-16-AID-Scarlet was observed in control, *gfp::rig-5*, *gfp::rig-5(ΔIg2)*, *gfp::rig-5(ΔIg3)* and *gfp::rig-5(ΔIg2-3)* following auxin-induced degradation in muscle cells. A schematic of RIG-5 functional domains is shown for each knock-in condition. Scale bar: 5 µm. **(b-c)** Quantification of ACR-16 (b) and RIG-5 (c) puncta density. Data are presented as boxplots showing lower and upper quartiles (box), mean (square), median (center line) and standard deviation (whiskers); the number of worms is indicated for each condition; Kruskal-Wallis and Dunn's test (b-c); ns: non-significant, *<0.05, ***<0.0005, ****<0.00005. Arrowheads delineate neurites of the ventral nerve cord where synapses form.

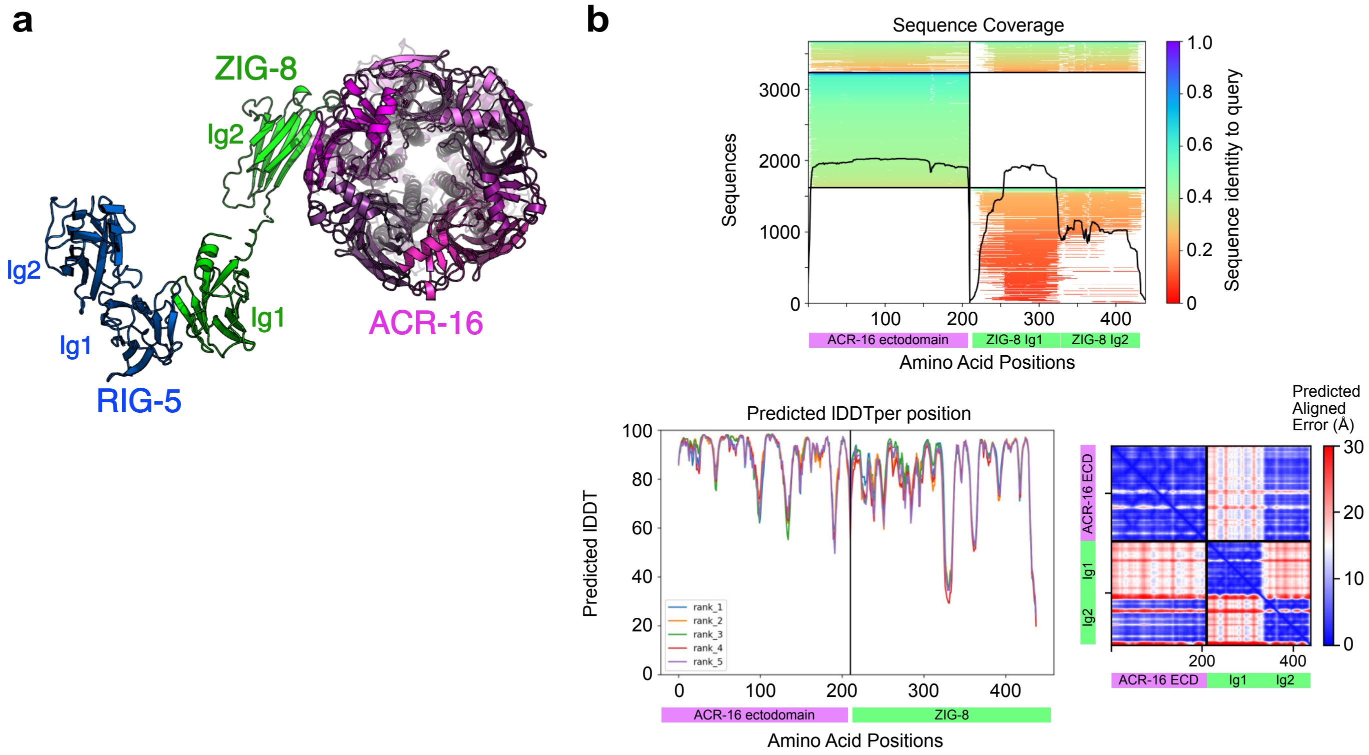

a
ZIG-8
Ig2
Ig2
Ig1
Ig1
ACR-16
RIG-5
b
Sequence Coverage
Sequences
Sequence identity to query
ACR-16 ectodomain
ZIG-8 Ig1
ZIG-8 Ig2
Amino Acid Positions
Predicted lDDTper position
Predicted lDDT
rank_1
rank_2
rank_3
rank_4
rank_5
ACR-16 ectodomain
ZIG-8
Amino Acid Positions
Predicted Aligned Error (Å)
ACR-16 ECD
Ig1
Ig2
ACR-16 ECD
Ig1
Ig2

Supplementary Figure 7 (related to Figure 5). Structure prediction of the RIG-5—ZIG-8—ACR-16 complex. (a) Structural model for the composite RIG-5—ZIG-8—ACR-16 complex with a top view showing the channel pore. **(b)** Colabfold plots for multiple sequence alignment sequence coverage, predicted local distance difference test (pLDDT), and predicted aligned error for the ACR-16 ectodomain complex with ZIG-8.

**Phenotype**	Wild-type locomotion	Intermediate locomotion	Paralyzed
**Strains**	Wild-type (N2 strain)<br>*acr-16(ok789)*<br>*acr-16(T201R)*	*unc-29(x29)*<br>*acr-16(T201R); unc-29(x29)*	*acr-16(ok789); unc-29(x29)*

Supplementary Figure 8 (related to Figure 5). The T201R mutation does not impair the function of ACR-16 receptors. Locomotion test of control, *acr-16(ok789), unc-29(x29), acr-16(T201R)* single mutants, as well as *acr-16(ok789); unc-29(x29)* and *acr-16(T201R); unc-29(x29)* double mutants.

Strain	Genotype	Figure
EN7643	*krSi81[Pmyo-3::TIR1::bfp] I; kr463[acr-16::aid::scarlet] V*	1, 3, Supp 3, 4
EN7973	*kr463[acr-16::aid::scarlet] V; krSi36[Prab-3::TIR1::bfp]*	1
EN7972	*krSi81[Pmyo-3::TIR1::bfp] I; krSi36[prab-3::TIR1::bfp] V; kr463[acr-16::aid::scarlet] V*	1
EN9486	*krSi81[Pmyo-3::TIR1::bfp] I; bab535[acr-16::aid::scarlet::spgfp11x3] V; krSi260[Ppept-3::cla-1::bfp]; krEx1453[Pflp-18::lox::STOP::lox::spgfp1-10; Pgap-14::cre; Pmyo-2::mCherry]*	1
EN9487	*krSi81[Pmyo-3::TIR1::bfp] I; bab535[acr-16::aid::scarlet::spgfp11x3] V; krSi260[Ppept-3::cla-1::bfp]; krEx1454[Pflp-18::lox::STOP::lox::spgfp1-10; Pgap-14::cre; Pmyo-2::mCherry]*	1
EN9488	*krSi81[Pmyo-3::TIR1::bfp] I; bab535[acr-16::aid::scarlet::spgfp11x3] V; krSi260[Ppept-3::cla-1::bfp]; krEx1455[Pflp-18::lox::STOP::lox::spgfp1-10; Pgap-14::cre; Pmyo-2::mCherry]*	1
EN9489	*krSi81[Pmyo-3::TIR1::bfp] I; bab535[acr-16::aid::scarlet::spgfp11x3] V; krSi260[Ppept-3::cla-1::bfp]; krEx1456[Pflp-18::lox::STOP::lox::spgfp1-10; Pgap-14::cre; Pmyo-2::mCherry]*	1
EN9480	*krSi81[Pmyo-3::TIR1::bfp] I; bab535[acr-16::aid::scarlet::spgfp11x3] V; krSi328[Punc-129::spgfp1-10]*	1
EN9481	*krSi81[Pmyo-3::TIR1::bfp] I; bab535[acr-16::aid::scarlet::spgfp11x3] V; krSi335[Punc-129::spgfp1-10]*	1
EN8784	*krSi81[Pmyo-3::TIR1::bfp] I; kr656[bfp::rig-5] I; kr643[gfp::zig-8] III; kr463[acr-16::aid::scarlet] V*	2, 5, Supp.4
EN8755	*krSi81[Pmyo-3::TIR1::bfp] I; bab446[spgfp11::rig-5] I; bab444[spgfp1-10::zig-8] III; kr463[acr-16::aid::scarlet] V*	2
EN8643	*krSi81[Pmyo-3::TIR1::bfp] I; kr643[gfp::zig-8] III; kr463[acr-16::aid::scarlet] V*	2, 3, Supp.4
EN8664	*krSi81[Pmyo-3::TIR1::bfp] I; rig-5(kr624) I; kr643[gfp::zig-8] III; kr463[acr-16::aid::scarlet] V*	2, Supp.4
EN8593	*krSi81[Pmyo-3::TIR1::bfp] I; kr640[gfp::rig-5] I; kr463[acr-16::aid::scarlet] V*	2, 3, Supp.4, 6
EN8632	*krSi81[Pmyo-3::TIR1::bfp] I; kr640[gfp::rig-5] I; zig-8(kr638) III; kr463[acr-16::aid::scarlet] V*	2, Supp.4
EN9065	*kr640[gfp::rig-5] I; kr653[scarlet::zig-8] III*	2
EN9048	*kr675[gfp::rig-5(F75D)] I; kr653[scarlet::zig-8] III*	2
EN9071	*kr640[gfp::rig-5] I; kr676[scarlet::zig-8(L77E)] III*	2
EN9049	*kr675[gfp::rig-5(F75D)] I; kr676[scarlet::zig-8(L77E)] III*	2
EN9193	*kr624(rig-5) I; kr653[scarlet::zig-8] III*	2
EN9194	*kr640[gfp::rig-5] I; kr638(zig-8) III*	2
EN8500	*krSi81[Pmyo-3::TIR1::bfp] I; rig-5(kr624) I; kr463[acr-16::aid::scarlet] V*	3, Supp.3, 4
EN8591	*krSi81[Pmyo-3::TIR1::bfp] I; zig-8(kr638) III; kr463[acr-16::aid::scarlet] V*	3, 5, Supp.3, 4
EN8741	*krSi81[Pmyo-3::TIR1::bfp] I; rig-5(kr624) I; zig-8(kr638) III; kr463[acr-16::aid::scarlet] V*	3

EN8916	*krSi81[Pmyo-3::TIR1::bfp] I; kr675[gfp::rig-5(F75D)] I; kr463[acr-16::aid::scarlet] V*	3
EN8748	*krSi81[Pmyo-3::TIR1::bfp] I; kr655[gfp::zig-8(L77E)] III; kr463[acr-16::aid::scarlet] V*	3
EN8633	*kr640[gfp::rig-5] I; acr-16(ok789) V*	3
EN8682	*kr643[gfp::zig-8] III; acr-16(ok789) V*	3
EN9139	*krSi81[Pmyo-3::TIR1::bfp] I; rig-5(kr624) I; kr463[acr-16::aid::scarlet] V; krEx1429[Ppept-3::bfp::rig-5; Pmyo-2::mCherry]*	4
EN8933	*krSi81[Pmyo-3::TIR1::bfp] I; zig-8(kr638) III; kr463[acr-16::aid::scarlet] V; krEx1399[Pflp-18::lox::STOP::lox::bfp::zig-8; Pgpa-14::cre; Pmyo-2::mCherry]*	4
EN9135	*krSi81[Pmyo-3::TIR1::bfp] I; rig-5(kr624) I; zig-8(kr638) III; kr463[acr-16::aid::scarlet] V; krEx1426[Pflp-18::lox::STOP::lox::gfp::zig-8; Ppept-3::bfp::rig-5; Pgpa-14::cre; Pmyo-2::mCherry]*	4
EN9136	*krSi81[Pmyo-3::TIR1::bfp] I; rig-5(kr624) I; zig-8(kr638) III; kr463[acr-16::aid::scarlet] V; krEx1427[Pflp-18::lox::STOP::lox::gfp::zig-8; Ppept-3::bfp::rig-5; Pgpa-14::cre; Pmyo-2::mCherry]*	4
EN9557	*rig-5(kr624) I; zig-8(kr638) III; bab462[cla-1::spgfp11x7] IV; krSi259[Ppept-3::spgfp1-10]; krEx1461[Pflp-18::lox::STOP::lox::scarlet::zig-8; Ppept-3::bfp::rig-5; Pgpa-14::cre; Pmyo-2::mCherry]*	4
EN9558	*rig-5(kr624) I; zig-8(kr638) III; bab462[cla-1::spgfp11x7] IV; krSi259[Ppept-3::spgfp1-10]; krEx1462[Pflp-18::lox::STOP::lox::scarlet::zig-8; Ppept-3::bfp::rig-5; Pgpa-14::cre; Pmyo-2::mCherry]*	4
EN9559	*rig-5(kr624) I; zig-8(kr638) III; bab462[cla-1::spgfp11x7] IV; krSi259[Ppept-3::spgfp1-10]; krEx1463[Pflp-18::lox::STOP::lox::scarlet::zig-8; Ppept-3::bfp::rig-5; Pgpa-14::cre; Pmyo-2::mCherry]*	4
EN9567	*rig-5(kr624) I; zig-8(kr638) III; bab462[cla-1::spgfp11x7] IV; krSi259[Ppept-3::spgfp1-10]; krEx1464[Pflp-18::lox::STOP::lox::bfp::rig-5; Ppept-3::scarlet::zig-8; Pgpa-14::cre; Pmyo-2::mCherry]*	4
EN9568	*rig-5(kr624) I; zig-8(kr638) III; bab462[cla-1::spgfp11x7] IV; krSi259[Ppept-3::spgfp1-10]; krEx1465[Pflp-18::lox::STOP::lox::bfp::rig-5; Ppept-3::scarlet::zig-8; Pgpa-14::cre; Pmyo-2::mCherry]*	4
EN9146	*krSi81[Pmyo-3::TIR1::bfp] I; kr656[bfp::rig-5] I; kr727[gfp::zig-8(∆Ig2)] III; kr463[acr-16::aid::scarlet] V*	5
EN9308	*krSi81[Pmyo-3::TIR1::bfp] I; kr656[bfp::rig-5] I; kr790[gfp::zig-8(∆Ig2)::CD4(Ig2)] III; kr463[acr-16::aid::scarlet] V*	5
EN9312	*krSi81[Pmyo-3::TIR1::bfp] I; kr656[bfp::rig-5] I; kr643[gfp::zig-8] III; kr793[acr-16(T201R)::aid::scarlet] V*	5
EN9246	*kr440[acr-16::scarlet] V; frm-3(gk585) X; krSi141[Punc-47::cla1::bfp]*	6
EN9243	*kr440[acr-16::scarlet] V; frm-3(gk585) X; krSi141[Punc-47::cla1::bfp]; krSi301[Pmyo-3::mNG::nlg-1(CLD)::zig-8(Ig2-GPI)]*	6
EN9331	*kr440[acr-16::scarlet] V; frm-3(gk585) X; krSi141[Punc-47::cla1::bfp]; krSi312[Pmyo-3::mNG::nlg-1(CLD)::zig-8(Ig1-GPI)]*	6
EN9435	*kr805[acr-16(T201R)::scarlet] V; frm-3(gk585) X; krSi141[unc-47-cla1-bfp]; krSi301[Pmyo-3::mNG::nlg-1(CLD)::zig-8(Ig2-GPI)]*	6
EN444	*kr440[acr-16::scarlet] V*	Supp.1

EN8343	*krSi80[Pmyo-3::TIR1::bfp] III; otIs669[NeuroPAL] V; krIs83[Pacr-16::sl2::AID::mNG]*	Supp.1
EN9002	*krSi81[Pmyo-3::TIR1::bfp] I; bab535[acr-16::aid::scarlet::spgfp11x3] V; krEx1404[Pflp-18::lox::STOP::lox::spgfp1-10; Pgap-14::cre; Pmyo-2::mCherry]*	1
EN9004	*krSi81[Pmyo-3::TIR1::bfp] I; bab535[acr-16::aid::scarlet::spgfp11x3] V; krEx1406[Pflp-18::lox::STOP::lox::spgfp1-10; Pgap-14::cre; Pmyo-2::mCherry]*	1
EN8778	*krSi81[Pmyo-3::TIR1::bfp] I; kr654[acr-16::aid::gfp] V*	1, Supp.1, Supp.3
EN9147	*krSi81[Pmyo-3::TIR1::bfp] I; bab462[cla-1::spgfp11x7] IV; kr463[acr-16::aid::scarlet] V; krEx1430[Plgc-55::lox::STOP::lox::spgfp1-10; Ptwk-40::cre; Pmyo-2::mCherry]*	Supp.1
EN7953	*krSi81[Pmyo-3::TIR1::bfp] I; krSi145[Punc-17::cla1::bfp] IV; kr463[acr-16::aid::scarlet] V*	Supp.1
EN8812	*krSi81[Pmyo-3::TIR1::bfp] I; rig-5(kr624) I; kr654[acr-16::aid::gfp] V*	Supp.3
EN8813	*krSi81[Pmyo-3::TIR1::bfp] I; zig-8(kr638) III; kr654[acr-16::aid::gfp] V*	Supp.3
EN8665	*krSi81[Pmyo-3::TIR1::bfp] I; kr463[acr-16::aid::scarlet] V; krEx1378[Pflp-18::lox::STOP::lox::sl2::gfp; Pgpa-14::cre; Pmyo-2::mCherry]*	Supp.5
EN8667	*krSi81[Pmyo-3::TIR1::bfp] I; rig-5(kr624) I; kr463[acr-16::aid::scarlet] V; krEx1378[Pflp-18::lox::STOP::lox::sl2::gfp; Pgpa-14::cre; Pmyo-2::mCherry]*	Supp.5
EN8680	*krSi81[Pmyo-3::TIR1::bfp] I; zig-8(kr638) III; kr463[acr-16::aid::scarlet] V; krEx1378[Pflp-18::lox::STOP::lox::sl2::gfp; Pgpa-14::cre; Pmyo-2::mCherry]*	Supp.5
EN8660	*kr463[acr-16::aid::scarlet] V; krSi241[Ppept-3::mNG]*	Supp.5
EN8661	*rig-5(kr624) I; kr463[acr-16::aid::scarlet] V; krSi241[Ppept-3::mNG]*	Supp.5
EN8763	*zig-8(kr638) III; kr463[acr-16::aid::scarlet] V; krSi241[Ppept-3::mNG]*	Supp.5
EN8732	*krSi81[Pmyo-3::TIR1::bfp] I; kr463[acr-16::aid::scarlet] V; krEx1382[Punc-119::mNG; Pmyo-2::mCherry]*	Supp.5
EN8734	*krSi81[Pmyo-3::TIR1::bfp] I; rig-5(kr624) I; kr463[acr-16::aid::scarlet] V; krEx1382[Punc-119::mNG; Pmyo-2::mCherry]*	Supp.5
EN8735	*krSi81[Pmyo-3::TIR1::bfp] I; zig-8(kr638) III; kr463[acr-16::aid::scarlet] V; krEx1382[Punc-119::mNG; Pmyo-2::mCherry]*	Supp.5
MCP518	*krSi81[Pmyo-3::TIR1::bfp] I; bab518[gfp::rig-5(ΔIg2)] I; kr463[acr-16::aid::scarlet] V*	Supp.6
MCP520	*krSi81[Pmyo-3::TIR1::bfp] I; bab520[gfp::rig-5(ΔIg3)] I; kr463[acr-16::aid::scarlet] V*	Supp.6
EN9123	*krSi81[Pmyo-3::TIR1::bfp] I; kr722[gfp::rig-5(ΔIg2-3)] I; kr463[acr-16::aid::scarlet] V*	Supp.6
N2 Bristol		Supp.8
RB918	*acr-16(ok789) V*	Supp.8
ZZ29	*unc-29(X29) I*	Supp.8
EN1258	*unc-29(X29) I; acr-16(ok789) V*	Supp.8
EN9533	*kr846 [acr-16(T201R)] V*	Supp.8
EN9534	*unc-29(X29) I; kr846 [acr-16(T201R)] V*	Supp.8

Supplementary Table 1. Strain list.

Allele name	Qualifiers	Gene locus	Chro-mo-some	Type	From
bab444	*[spgfp1-10::zig-8]*	*zig-8*	III	CRISPR/Cas9	Segicel
bab446	*[spgfp11::rig-5]*	*rig-5*	I	CRISPR/Cas9	Segicel
bab462	*[cla-1::spgfp11x7]*	*cla-1*	IV	CRISPR/Cas9	Segicel
bab518	*[gfp::rig-5(ΔIg2)]* deletion R144-G182 included	*rig-5*	I	CRISPR/Cas9	Segicel
bab520	*[gfp::rig-5(ΔIg3)]* deletion S234-I323 included	*rig-5*	I	CRISPR/Cas9	Segicel
bab526	*[gfp::rig-5(ΔIg1)]* deletion M31-K128 included	*rig-5*	I	CRISPR/Cas9	Segicel
bab528	*[gfp::zig-8(ΔIg1)]* deletion Q42-E66 included	*zig-8*	III	CRISPR/Cas9	Segicel
bab535	*[acr-16::aid::scarlet::gfp11x3]*	*acr-16*	V	CRISPR/Cas9	Segicel
gk585		*frm-3*	X		*C. elegans* Gene Knockout Consortium
kr440	*[acr-16::scarlet]*	*acr-16*	V	CRISPR/Cas9	Bessereau lab
kr463	*[acr-16::aid::scarlet]*	*acr-16*	V	CRISPR/Cas9	Bessereau lab
kr573	*[rig-5(Q16*)]*	*rig-5*	I	EMS mutant	Bessereau lab
kr624	deletion from ATG to stop included	*rig-5*	I	CRISPR/Cas9	Bessereau lab
kr638	deletion from ATG to stop included	*zig-8*	III	CRISPR/Cas9	Bessereau lab
kr640	*[gfp::rig-5]*	*rig-5*	I	CRISPR/Cas9	Bessereau lab
kr643	*[gfp::zig-8]*	*zig-8*	III	CRISPR/Cas9	Bessereau lab
kr653	*[scarlet::zig-8]*	*zig-8*	III	CRISPR/Cas9	Bessereau lab
kr654	*[acr-16::aid::gfp]*	*acr-16*	V	CRISPR/Cas9	Bessereau lab
kr655	*[gfp::zig-8(L77E)]*	*zig-8*	III	CRISPR/Cas9	Bessereau lab
kr656	*[bfp::rig-5]*	*rig-5*	I	CRISPR/Cas9	Bessereau lab
kr675	*[gfp::rig-5(F75D)]*	*rig-5*	I	CRISPR/Cas9	Bessereau lab

kr676	*[scarlet::zig-8(L77E)]*	*zig-8*	III	CRISPR/Cas9	Bessereau lab
kr722	*[gfp::rig-5(ΔIg2-3)]* deletion R144-G182 and S234-I323 included	*rig-5*	I	CRISPR/Cas9	Bessereau lab
kr727	*[gfp::zig-8(ΔIg2)]* deletion S149-I188 and K195-N240 included	*zig-8*	III	CRISPR/Cas9	Bessereau lab
kr790	*[gfp::zig-8(ΔIg2)-CD4(Ig2)]*	*zig-8*	III	CRISPR/Cas9	Bessereau lab
kr793	*[acr-16(T201R)::aid::scarlet]*	*acr-16*	V	CRISPR/Cas9	Bessereau lab
kr805	*[acr-16(T201R)::scarlet]*	*acr-16*	V	CRISPR/Cas9	Bessereau lab
kr846	*[acr-16(T201R)]*	*acr-16*	V	CRISPR/Cas9	Bessereau lab
kr849	*[rig-5(P131S)]*	*rig-5*	I	EMS mutant	Bessereau lab
kr850	*[zig-8(Q92*)]*	*zig-8*	III	EMS mutant	Bessereau lab
kr851	*[zig-8(A68T)]*	*zig-8*	III	EMS mutant	Bessereau lab
kr852	*[zig-8(I239F)]*	*zig-8*	III	EMS mutant	Bessereau lab
krEx1378	*[Pflp-18::lox::STOP::lox::sl2::gfp; Pgpa-14::cre; Pmyo-2::mCherry]*			Extrachromosomal array	Bessereau lab
krEx1382	*[Punc-129::mNG;Pmyo-2::mCherry]*			Extrachromosomal array	Bessereau lab
krEx1399	*[Pflp-18::lox::STOP::lox::bfp::zig-8; Pgpa-14::cre; Pmyo-2::mCherry]*			Extrachromosomal array	Bessereau lab
krEx1404	*[Pflp-18::lox::STOP::lox::spgfp1-10; Pgpa-14::cre; Pmyo-2::mCherry]*			Extrachromosomal array	Bessereau lab
krEx1406	*[Pflp-18::lox::STOP::lox::spgfp1-10; Pgpa-14::cre; Pmyo-2::mCherry]*			Extrachromosomal array	Bessereau lab
krEx1426	*[Pflp-18::lox::STOP::lox::gfp::zig-8; Ppept-3::bfp::rig-5; Pgpa-14::cre; Pmyo-2::mCherry]*			Extrachromosomal array	Bessereau lab

krEx1427	*[Pflp-18::lox::STOP::lox::gfp::zig-8; Ppept-3::bfp::rig-5; Pgpa-14::cre; Pmyo-2::mCherry]*			Extrachromosomal array	Bessereau lab
krEx1429	*[Ppept-3::bfp::rig-5; Pmyo-2::mCherry]*			Extrachromosomal array	Bessereau lab
krEx1430	*[Plgc-55::lox::STOP::lox::spgfp1-10; Ptwk-40::cre; Pmyo-2::mCherry]*			Extrachromosomal array	Bessereau lab
krEx1453	*[Pflp-18::lox::STOP::lox::spgfp1-10; Pgpa-14::cre; Pmyo-2::mCherry]*			Extrachromosomal array	Bessereau lab
krEx1454	*[Pflp-18::lox::STOP::lox::spgfp1-10; Pgpa-14::cre; Pmyo-2::mCherry]*			Extrachromosomal array	Bessereau lab
krEx1455	*[Pflp-18::lox::STOP::lox::spgfp1-10; Pgpa-14::cre; Pmyo-2::mCherry]*			Extrachromosomal array	Bessereau lab
krEx1456	*[Pflp-18::lox::STOP::lox::spgfp1-10; Pgpa-14::cre; Pmyo-2::mCherry]*			Extrachromosomal array	Bessereau lab
krEx1461	*[Pflp-18::lox::STOP::lox::scarlet::zig-8; Ppept-3::bfp::rig-5; Pgpa-14::cre; Pmyo-2::mCherry]*			Extrachromosomal array	Bessereau lab
krEx1462	*[Pflp-18::lox::STOP::lox::scarlet::zig-8; Ppept-3::bfp::rig-5; Pgpa-14::cre; Pmyo-2::mCherry]*			Extrachromosomal array	Bessereau lab
krEx1463	*[Pflp-18::lox::STOP::lox::scarlet::zig-8; Ppept-3::bfp::rig-5; Pgpa-14::cre; Pmyo-2::mCherry]*			Extrachromosomal array	Bessereau lab
krEx1464	*[Pflp-18::lox::STOP::lox::bfp::rig-5; Ppept-3::scarlet::zig-8; Pgpa-14::cre; Pmyo-2::mCherry]*			Extrachromosomal array	Bessereau lab

krEx1465	*[Pflp-18::lox::STOP::lox::bfp::rig-5; Ppept-3::scarlet::zig-8; Pgpa-14::cre; Pmyo-2::mCherry]*			Extrachromosomal array	Bessereau lab
krIs83	*[Pacr-16::sl2::AID::mNG]*			X-ray insertion	Bessereau lab
krSi141	*[Punc-47::cla1::bfp]*			Minimos insertion	Bessereau lab
krSi145	*[Punc-17::cla1::bfp]*		IV	Minimos insertion	Bessereau lab
krSi241	*[Ppept-3::mNG]*			Minimos insertion	Bessereau lab
krSi259	*[Ppept-3::spgfp1-10]*			Minimos insertion	Bessereau lab
krSi260	*[Ppept-3::spgfp1-10]*			Minimos insertion	Bessereau lab
krSi301	*[Pmyo-3::mNG::nlg-1(CLD)::zig-8(Ig2-GPI)]*			Minimos insertion	Bessereau lab
krSi312	*[Pmyo-3::mNG::nlg-1(CLD)::zig-8(Ig1-GPI)]*			Minimos insertion	Bessereau lab
krSi328	*[Punc-129::spgfp1-10]*			Minimos insertion	Bessereau lab
krSi335	*[Punc-129::spgfp1-10]*			Minimos insertion	Bessereau lab
krSi36	*[Prab-3::TIR1::bfp]*		V	Minimos insertion	Zhou et al, 2021
krSi80	*[Pmyo-3::TIR1::bfp]*		III	Minimos insertion	Bessereau lab
krSi81	*[Pmyo-3::TIR1::bfp]*		I	Minimos insertion	Bessereau lab
ok789			V		*C. elegans* Gene Knockout Consortium
otIs669	*[NeuroPAL]*		V		Yemini et al., 2021[2]
x29		*unc-29*	I		CGC

Supplementary Table 2. Allele list.

crRNA name	Sequence	Alleles
crMM06	TCGGGAGCACCTACCTTCAG	*kr624*
crMM07	acaaaaaggcataaaaattg	*kr624*
crMM10	Ttgatggttgttgaattgtt	*kr640, kr656, bab446*
crMM12	catttggacaccgccgacgg	*kr638*
crMM13	tagttaattttcagatgttc	*kr638*
crMM14	ATGCACCATGTCctgcaaat	*kr643, kr653, bab444*
crMM17	gcagtttTCAGTTTATTGCA	*bab462*
crMM18	TGGGTTGACAAGTGTGAGCA	*kr654*
crMM19	AGCTGTCAATAAGGCTCCGT	*kr655, kr676*
crMM20	TCGTCGAACGAGAGCAGCCG	*kr675*
crMM27	tctcaacttcccctacaact	*kr727*
crMM37	GTGTCATTAAAGTTTTTTTc	*kr790*
crMM38	atgaaactcccatattcagt	*kr790*
crMM39	aaagtttacagTGACAACTG	*kr793, kr805, kr846*

Supplementary Table 3. List of guides used for CRISPR/Cas9 gene editing.

Name	Description	Alleles
pMM04	*Pflp-18::lox::STOP::lox::sl2::gfp*	*krEx1378*
pMM06	*Ppept-3::mNG*	*krSi241*
pMM12	*Punc-129::mNG*	*krEx1382*
pMM13	*Ppept-3::spgfp1-10*	*krSi259, krSi260*
pMM15	*Pflp18::lox::STOP::lox::spgfp1-10*	*krEx1404, krEx1406, krEx1453, krEx1454, krEx1455, krEx1456*
pMM19	*Ppept-3::bfp::rig-5*	*krEx1426, krEx1427, krEx1429, krEx1461, krEx1462, krEx1463*
pMM20	*Pflp18::lox::STOP::lox::bfp::rig-5*	*krEx1464, krEx1465*
pMM23	*Pflp18::lox::STOP::lox::bfp::zig-8*	*krEx1399*
pMM31	*Ptwk-40-cre*	*krEx1430*
pMM34	*Pflp18::lox::STOP::lox::gfp::zig-8*	*krEx1426, krEx1427*
pMM36	*Plgc-55::lox::STOP::lox::spgfp1-10*	*krEx1430*
pMM41	*Pmyo-3::mNG::nlg-1(CLD)::zig-8(Ig2-GPI)*	*krSi301*
pMM51	*Pmyo-3::mNG::nlg-1(CLD)::zig-8(Ig1-GPI)*	*krSi312*
pMM55	*Ppept-3::scarlet::zig-8*	*krEx1464, krEx1465*
pMM56	*Pflp-18::lox::STOP::lox::scarlet::zig-8*	*krEx1461, krEx1462, krEx1463*
pLP04	*Punc-129::spgfp1-10*	*krSi328, krSi335*
pNP259	*Pgpa-14::cre*	*krEx1378, krEx1399, krEx1404, krEx1406, krEx1426, krEx1427, krEx1453, krEx1454, krEx1455, krEx1456,krEx1461, krEx1462, krEx1463, krEx1464, krEx1465*
pCFJ90	*Pmyo-2::mCherry*	all krEx alleles

Supplementary Table 4. Plasmid list.

Promoters	Froward Primer (5' to 3')	Reverse Primer (5' to 3')
Pmyo-3	CGGCTATAATAAGTTCTTG	TCTAGATGGATCTAGTGGTCG
Punc-17	tacaccaatcatttctcccc	ttttgaacaagagatgcgg
Punc-129	GGGAAACATGATATCGACGG	TTTCTTGCTTGCTCTTCCAA
Ppept-3	GATAAAAATAACAGAATTAG	CGGGAGAGGCGGAATTAATTTTATAG
Pflp-18	CTGTCACATACTGCTCGAATCG	TCTAACCCTGAAATTATTAT
Pgpa-14	ACGACGACAAGAAGGTAATT	GGGAGAAGCCCGGAGGTAC
Ptwk-40	tctaatcactatcacgtggg	tgaatattcatcactcgata
Plgc-55	tgttgcttttcttctcctca	ttcatttcgacatctatttg

Supplementary Table 5. List of primers used to amplify promoters.

Alleles	Injection mix
krEx1453, krEx1454, krEx1455, krEx1456	2.5 ng/µL pCFJ90 (*Pmyo-2::mCherry*), 50 ng/µL pMM15 (*Pflp18::lox::STOP::lox::spgfp1-10*) and 50 ng/µL pNP259 (*Pgpa-14::cre*)
krEx1429	2.5 ng/µL pCFJ90 (*Pmyo-2::mCherry*), 50 ng/µL pMM19 (*Ppept-3::bfp::rig-5*) and 50 ng/µL 1kb$^+$ Invitrogen
krEx1399	2.5 ng/µL pCFJ90 (*Pmyo-2::mCherry*), 50 ng/µL pMM23 (*Pflp-18::lox::STOP::lox::bfp::zig-8*) and 50 ng/µL pNP259 (*Pgpa-14::cre*)
krEx1426, krEx1427	2.5 ng/µL pCFJ90 (*Pmyo-2::mCherry*), 30 ng/µL pMM19 (*Ppept-3::bfp::rig-5*), 30 ng/µL pMM34 (*Pflp-18::lox::STOP::lox::gfp::zig-8*), and 30 ng/µL pNP259 (*Pgpa-14::cre*)
krEx1461, krEx1462, krEx1463	2.5 ng/µL pCFJ90 (*Pmyo-2::mCherry*), 30 ng/µL pMM19 (*Ppept-3::bfp::rig-5*), 25 ng/µL pMM56 (*Pflp-18::lox::STOP::lox::scarlet::zig-8*), and 30 ng/µL pNP259 (*Pgpa-14::cre*)
krEx1464, krEx1465	2.5 ng/µL pCFJ90 (*Pmyo-2::mCherry*), 30 ng/µL pMM20 (*Pflp18::lox::STOP::lox::bfp::rig-5*), 25 ng/µL pMM55 (*Ppept-3::scarlet::zig-8*), and 30 ng/µL pNP259 (*Pgpa-14::cre*)
krEx1404, krEx1406	2.5 ng/µL pCFJ90 (*Pmyo-2::mCherry*), 50 ng/µL pMM15 (*Pflp18::lox::STOP::lox::spgfp1-10*) and 50 ng/µL pNP259 (*Pgpa-14::cre*)
krEx1430	2.5 ng/µL pCFJ90 (*Pmyo-2::mCherry*), 50 ng/µL pMM36 (*Plgc-55::lox::STOP::lox::spgfp1-10*) and 50 ng/µL pMM31 (*twk-40::cre*)
krEx1378	2.5 ng/µL pCFJ90 (*Pmyo-2::mCherry*), 50 ng/µL pMM04 (*Pflp-18::lox::STOP::lox::sl2::gfp*) and 50 ng/µL pNP259 (*Pgpa-14::cre*)
krEx1382	2.5 ng/µL pCFJ90 (Pmyo-2::mCherry), 50 ng/µL pMM12 (Punc-129::mNG) and 50 ng/µL 1kb+ Invitrogen

Supplementary Table 6. Composition of the injection mix used to generate extrachromosomal array lines.

Protein	Sequence used in this study	Wormbase transcript	Uniprot accession number
RIG-5	MYLFALLCGVLLVFKQACSRGAPPTIQQPSMSSAVALLGQ<br>DVDFTCIVNDLGSHMVAFVKADSPPRLLSFDEKVFRRRNK<br>YELKPRIGDLHNEWVLTIKNVQESDRGNYSCQINTEPITLS<br>TGELDVKVPPVVSRSTPAAVEVREGNNVSLTCKADGNPTP<br>TVIWRRQDRQIIRYNGATGFGASVFHGPVLHLTKVSRKH<br>MSEYLCVASNGIPPDESWTVKLLVTFPPLVQAQSETVQAS<br>VGSMARMVCTTEAWPRPEMGWEKDGEPVYESNNVAM<br>THTVSGQYHSVHILEIRNVQSSHFGVYRCVAKNDNGIHHS<br>QVTLNQISHNHFTNSNLIPEGSGMPRGYSEDDEADEEED<br>NLENQKNDSEEEDSQSPPQLEFYSQARRHESRNAPTTRSE<br>LRAIQVDSASFPSLLLITIIYFVYHGF	C36F7.4e.1 with the 60 first codons removed	none
ZIG-8	MRRFSNICVILFSFLYATGHGASEEVMACLRQERSRVENPS<br>QTIVNVVAENPAYLHCSVPPDAEHEIAWTRVSDGALLTAG<br>NRTFTRDPRWQVSKKSANIWVLNLRRAEQQDSGCYLCEI<br>NDKHNTVYAVYLKVLEPPLPSPSSLQKKSTKLMANMSGDE<br>VVLNCTVTSTDKDEEVLDVVWTRDGNTINFNDTEKYILKV<br>KRDAGVVIETMRIRKATMEDDGNYACEHSQQKASQIVHI<br>NKAEAQTSNSATFPCSIFSISIFMYFLYL	Y39E4B.8.1	G5ED00
ACR-16	MSVCTLLISCAILAAPTLGSLQERRLYEDLMRNYNNLERPV<br>ANHSEPVTVHLKVALQQIIDVDEKNQVVYVNAWLDYTW<br>NDYNLVWDKAEYGNITDVRFPAGKIWKPDVLLYNSVDTN<br>FDSTYQTNMIVYSTGLVHWVPPGIFKISCKIDIQWFPFDEQ<br>KCFFKFGSWTYDGYKLDLQPATGGFDISEYISNGEWALPLT<br>TVERNEKFYDCCPEPYPDVHFYLHMRRRTLYYGFNLIMPCI<br>LTTLMTLLGFTLPPDAGEKITLQITVLLSICFFLSIVSEMSPPT<br>SEAVPLLGIFFTCCMIVVTASTVFTVYVLNLHYRTPETHDM<br>GPWTRNLLLYWIPWILRMKRPGHNLTYASLPSLFSTKPNR<br>HSESLIRNIKDNEHSLSRANSFDADCRLNQYIMTQSVSNGL<br>TSLGSIPSTMISSNGTTTDVSQQATLLILHRIYHELKIVTKR<br>MIEGDKEEQACNNWKFAAMVVDRLCLYVFTIFIIVSTIGIF<br>WSAPYLVA	F25G6.3.1	P48180

Supplementary table 7. Protein sequences of RIG-5, ZIG-8 and ACR-16 used in this study.

SUPPLEMENTARY REFERENCES

1. Cheng, S. *et al.* Family of neural wiring receptors in bilaterians defined by phylogenetic, biochemical, and structural evidence. *Proc National Acad Sci* **116**, 201818631 (2019).

2. Yemini, E. *et al.* NeuroPAL: A Multicolor Atlas for Whole-Brain Neuronal Identification in C. elegans. *Cell* **184**, 272-288.e11 (2021).
